# The M1311V variant of ATP7A is associated with impaired trafficking and copper homeostasis in models of motor neuron disease

**DOI:** 10.1016/j.nbd.2020.105228

**Published:** 2020-12-24

**Authors:** Nadine Bakkar, Alexander Starr, Benjamin E. Rabichow, Ileana Lorenzini, Zachary T. McEachin, Robert Kraft, Matthew Chaung, Sam Macklin-Isquierdo, Taylor Wingfield, Briggs Carhart, Nathan Zahler, Wen-Hsuan Chang, Gary J. Bassell, Alexandre Betourne, Nicholas Boulis, Samuel V. Alworth, Justin K. Ichida, Paul R. August, Daniela C. Zarnescu, Rita Sattler, Robert Bowser

**Affiliations:** a Department of Neurobiology, Barrow Neurological Institute, Phoenix, AZ 85013, USA; b Department of Cell Biology, Emory University School of Medicine, Atlanta, GA 30322, USA; c Departments of Molecular and Cellular Biology, Neuroscience, and Neurology, University of Arizona, Tucson, AZ 85721, USA; d Icagen Inc., Oro Valley, AZ 85755, USA; e AcuraStem Incorporated, Monrovia, CA 91016, USA; f Above and Beyond NB, LLC, Atlanta, GA 30309, USA; g Department of Neurosurgery, Emory University School of Medicine, Atlanta, GA 30322, USA

**Keywords:** Copper transport, Amyotrophic lateral sclerosis, ATP7A, iPSC derived motor neurons, Intracellular transport, Copper toxicity

## Abstract

Disruption in copper homeostasis causes a number of cognitive and motor deficits. Wilson’s disease and Menkes disease are neurodevelopmental disorders resulting from mutations in the copper transporters ATP7A and ATP7B, with ATP7A mutations also causing occipital horn syndrome, and distal motor neuropathy. A 65 year old male presenting with brachial amyotrophic diplegia and diagnosed with amyotrophic lateral sclerosis (ALS) was found to harbor a p.Met1311Val (M1311V) substitution variant in ATP7A. ALS is a fatal neurodegenerative disease associated with progressive muscle weakness, synaptic deficits and degeneration of upper and lower motor neurons. To investigate the potential contribution of the ATP7A^M1311V^ variant to neurodegeneration, we obtained and characterized both patient-derived fibroblasts and patient-derived induced pluripotent stem cells differentiated into motor neurons (iPSC-MNs), and compared them to control cell lines. We found reduced localization of ATP7A^M1311V^ to the trans-Golgi network (TGN) at basal copper levels in patient-derived fibroblasts and iPSC-MNs. In addition, redistribution of ATP7A^M1311V^ out of the TGN in response to increased extracellular copper was defective in patient fibroblasts. This manifested in enhanced intracellular copper accumulation and reduced survival of ATP7A^M1311V^ fibroblasts. iPSC-MNs harboring the ATP7A^M1311V^ variant showed decreased dendritic complexity, aberrant spontaneous firing, and decreased survival. Finally, expression of the ATP7A^M1311V^ variant in *Drosophila* motor neurons resulted in motor deficits. Apilimod, a drug that targets vesicular transport and recently shown to enhance survival of C9orf72-ALS/FTD iPSC-MNs, also increased survival of ATP7A^M1311V^ iPSC-MNs and reduced motor deficits in Drosophila expressing ATP7A^M1311V^. Taken together, these observations suggest that ATP7A^M1311V^ negatively impacts its role as a copper transporter and impairs several aspects of motor neuron function and morphology.

## 1. Introduction

Cellular copper (Cu) homeostasis is essential for normal metabolic functions including cellular respiration, neuropeptide processing, and iron transport (Bhattacharjee et al., 2016). Tightly regulated copper metabolism is especially vital in the central nervous system (CNS), where copper is critical to support synapse function and modulate long term potentiation, amyloid precursor protein (APP) trafficking as well as matrix metalloprotease 9 (MMP9) and AMP-activated protein kinase (AMPK) activation (Acevedo et al., 2011; Bhattacharjee et al., 2016; Gybina and Prohaska, 2008; Hwang et al., 2007; Lutsenko et al., 2010; Opazo et al., 2014; Scheiber et al., 2014). Copper is a trace element required for the activity of a number of essential enzymes, with the size of the copper proteome being close to 1% of the total proteome in eukaryotes (Andreini et al., 2008). Excess copper is, however, toxic. Therefore, a tight regulation of Cu balance exists in cells, mediated by copper transporting ATPase’s such as ATP7A and ATP7B, copper uptake protein 1 (CTR1), and multiple cytosolic copper chaperones (Lutsenko et al., 2010).

**Fig. 1.**
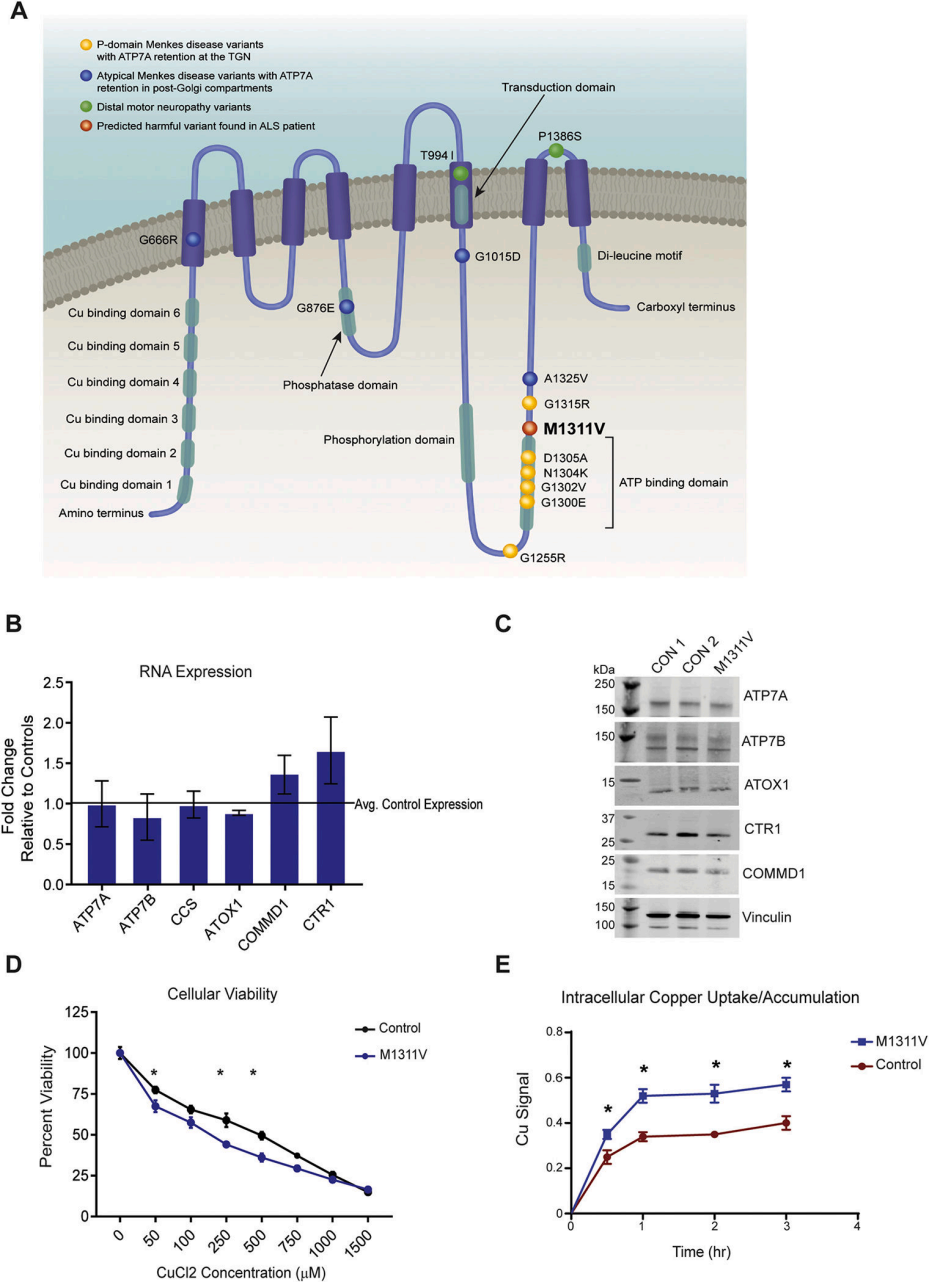
A) Schematic showing the structure of the ATP7A protein, and the location of some of the pathogenic mutations previously reported and of the M1311V variant. B) Total RNA was prepared from two control and one ATP7A^M1311V^ line and mRNA levels of the indicated genes were measured, normalized to GAPDH. C) Total protein lysates were prepared from two control and the ATP7A^M1311V^ fibroblast lines, separated on gels and western blots performed for the indicated proteins. Vinculin was used as a loading control. D) Two control cell lines and the ATP7A^M1311V^ fibroblasts were seeded at a density of 3000 cells/well. The next day, cells were treated with serial dilutions of CuCl_2_ for 24 h, before the MTT reagent was added. The reaction was allowed to develop for 3 h at 37 °C, and absorbances were measured. Each time point was an average of quadruplicate readings and values in untreated wells for each lines were made to be 100%. Asterisks denote significance, with *p*-values of 0.0441, 0.0012 and 0.0038 at the 50, 250 and 500uM CuCl2 concentrations, respectively. E) Intracellular Cu uptake/accumulation in control and ATP7A^M1311V^ fibroblasts exposed to 250uM CuCl2 for various times. Results were normalized to cellular density. Error bars show the standard deviation of four technical replicate measurements from one representative experiment. The experiment was repeated 3 times with similar results showing an M1311v/control intracellular Cu^2+^ ratios of 1.5 ± 0.3; 1.5 ± 0.1 and 1.5 ± 0.2.

ATP7A, also known as Menkes protein (MNK), is a multispan membrane protein that contains eight transmembrane domains and six copper binding motifs (Fig. 1A). ATP7A normally resides in the trans-Golgi apparatus (TGN) where it functions to supply Cu to newly synthesized cuproenzymes such as dopamine β-hydroxylase, peptidylglycine α-amidating monooxygenase, lysyl amine oxidase, and cytochrome *c* oxidase (Lutsenko et al., 2007). ATP7A accepts copper atoms from the copper chaperone Antioxidant 1 Copper Chaperone (ATOX1) and functions to pump this element across biological membranes into the Golgi lumen using the energy of ATP hydrolysis (Voskoboinik et al., 1998). When Cu is in excess, ATP7A undergoes copper-stimulated redistribution from the TGN to the plasma membrane, pumping copper out of the cell, thus maintaining intracellular copper homeostasis. ATP7A then recycles back to the TGN, a trafficking process that requires di-leucine motifs in its C terminus and is regulated by clathrin and other adapter protein complexes including AP1, AP2, and Rab22 (Holloway et al., 2013; Pase et al., 2004; Petris et al., 1996; Yi and Kaler, 2015).

ATP7A is ubiquitously expressed in the body. In the CNS, *Atp7a* is more highly expressed in the brain barriers, the brain capillaries and choroid plexus, than in brain parenchyma (Choi and Zheng, 2009). Within the parenchyma itself, higher levels of the ATP7A transporter reside in pyramidal neurons and hippocampal interneurons (Schlief et al., 2005), and stimulation of the NMDA receptor result in its trafficking out of the TGN and into dendrites (Dodani et al., 2011). *Atp7a* expression has also been detected in murine astrocytes (Scheiber et al., 2012), activated microglia surrounding Aβ plaques (Zheng et al., 2010) and cerebellar Purkinje neurons (Davies et al., 2013). Genetic variants of ATP7A are responsible for multiple diseases with neurologic and neuromuscular symptoms including Menkes disease (MD; OMIM 309400), occipital horn syndrome (OHS; OMIM 304150), and X-linked distal motor neuropathy (SMAX3, OMIM 300489) (Kaler, 2011; Tumer, 2013). A number of ATP7A variants cause MD through reduction in protein levels, function, or ATP7A trafficking, resulting in decreased copper in the serum and brain, and decreased activity of essential cuproenzymes (Menkes et al., 1962). OHS is a neurologically milder variant of MD with generalized muscle weakness and low to normal serum copper levels (Kaler, 1994; Kaler et al., 1994). SMAX3 exhibits no overt copper metabolic disturbances but is associated with progressive distal motor neuropathy and distal degeneration of axons that spreads to neuronal cell bodies, implicating ATP7A in spinal motor neurons and synaptic function (Kennerson et al., 2010). In fact, deletion of ATP7A in spinal motor neurons of mice results in progressive deterioration of gait, age-dependent muscle atrophy, denervation of neuromuscular junctions, and a loss of motor neuron cell bodies, highlighting the essential roles for ATP7A and copper in the maintenance and function of motor neurons (Hodgkinson et al., 2015a; Hodgkinson et al., 2015b). In addition, an ATP7A variant that results in the distal neuropathy phenotype was recently found to disrupt its interaction with p97/VCP, an ATPase involved in protein degradation which is mutated in motor neuron diseases such as amyotrophic lateral sclerosis (ALS), inclusion body myopathy with early -onset Paget disease and frontotemporal dementia (IBMPFD), and Charcot -Marie -Tooth disease type 2Y (Gonzalez et al., 2014; Johnson et al., 2010; Watts et al., 2007; Yi and Kaler, 2018). ATP7A disease-causing mutations are distributed throughout the protein, with some clustering within or near the functional domains of the protein (Fig. 1A).

A variant of ATP7A, ATP7A^M1311V^ has recently been identified in a patient with brachial amyotrophic diplegia/flail arm syndrome (also called man-in-barrel syndrome) with slow-progressing ALS (Yun et al., 2020). ALS is a neurodegenerative disease characterized by loss of motor neurons in the brain, brainstem and spinal cord, along with muscle atrophy (Al-Chalabi et al., 2012). ALS has multiple variants and clinical presentations, with the more classical presentations being bulbar and limb-onset ALS while some rare variants of ALS affect isolated spinal regions (Jawdat et al., 2015). The ATP7A^M1311V^ variant is located within the P-domain that has a remarkable clustering of disease-associated variants of ATP7A (Fig. 1A). Indeed, mutations at residues 1300, 1302, 1304, 1305 and 1315 result in classical Menkes disease with TGN retention, while a mutation at residue 1325 is associated with atypical Menkes disease and post-Golgi compartment retention (Skjorringe et al., 2017) with two additional potential disease-causing missense variants are located at residues 1335 and 1338 (Kaler et al., 2020). Patient-derived induced pluripotent stem cells (iPSC) carrying ATP7A^M1311V^ differentiated into motor neurons showed elevated levels of reactive oxygen species and cell damage markers (lactate dehydrogenase) as well as decreased firing action potential compared to controls. These phenotypes could be rescued with CRISPR-mediated correction of the variant, thus linking the variant to impaired motor neuron function and potentially neurodegeneration (Yun et al., 2020).

To further investigate how this mutation impacts motor neuron function or may contribute to neurodegeneration, we studied the expression of ATP7A^M1311V^ in patient-derived cells and found reduced TGN localization of ATP7A^M1311V^ protein at basal copper levels in both patient fibroblasts and induced pluripotent stem cells differentiated into motor neurons (iPSC-MNs). These cells also failed to relocate ATP7A back to the TGN following copper treatment and washout. In addition, ATP7A^M1311V^ iPSC-MNs exhibited decreased dendritic complexity and aberrant spontaneous firing when compared to iPSC-MNs expressing wildtype ATP7A, as well as decreased neuronal survival following growth factor withdrawal suggesting impaired neuronal functions and increased susceptibility to cellular stressors. To further test the ability of the ATP7A^M1311V^ variant to cause motor neuron dysfunction we generated a Drosophila model of disease based on overexpression of ATP7A^M1311V^ in neurons or glia. These experiments showed that ATP7A^M1311V^ causes significant locomotor deficits in larvae. Collectively, our findings in multiple models indicate that the M1311V variant of ATP7A results in altered sub-cellular distribution and function of ATP7A leading to aberrant intracellular copper accumulation, as well as reduced neuronal dendritic complexity, functionality and viability of iPSC derived motor neurons, likely contributing to ALS disease pathogenesis.

## 2. Results

### 2.1. Increased Cu uptake and reduced survival of ATP7A^M1311V^ patient derived fibroblasts

Initial attempts to understand the effects of the ATP7A^M1311V^ variant utilized patient-derived fibroblasts to explore gene expression and protein levels of the variant. Given that classical Menkes disease is often associated with gross changes in ATP7A expression, we quantified mRNA (Fig. 1B) and protein (Fig. 1C, S1A) levels of ATP7A. The ATP7A protein was detected at the expected molecular weight in the ATP7A^M1311V^ fibroblasts, and its protein and mRNA levels were comparable to two healthy control fibroblast lines, suggesting no variant-induced impact effect on expression, stability or splicing of ATP7A. No changes were observed in the mRNA or protein levels of other genes involved in copper metabolism, including: ATP7B, the copper chaperone for superoxide dismutase (CCS), antioxidant protein 1 (ATOX1, the metallochaperone responsible for transporting cytosolic copper to ATP7A and ATP7B), copper metabolism MURR domain 1 (COMMD1, an ATP7A binding partner), and the high affinity copper uptake protein 1 (CTR1), the primary controller of copper influx (Fig. 1B, C and S1A). Consistent with increased reactive oxygen species previously described in neural progenitor cells (Yun et al., 2020), we observed a significantly decreased ratio of reduced to oxidized glutathione (GSH/GSSG) in fibroblasts carrying the M1311V mutation compared to two control lines, reflecting an inherent increased state of intracellular oxidative stress (Fig. S1B).

We next determined whether ATP7A^M1311V^ fibroblasts are more vulnerable to copper exposure, given the role of ATP7A in the regulation of copper homeostasis. We treated two control fibroblast lines and the ATP7A^M1311V^ fibroblasts with increasing concentrations of CuCl_2_ for 24 h and assayed the number of viable cells using the MTT assay. While all fibroblast lines were resistant to short-term Cu exposure (2 and 4 h, data not shown), ATP7A^M1311V^ cells were more vulnerable to 24 h Cu treatment. CuCl_2_ concentrations between 50 and 500 μM triggered significantly reduced cellular viability when compared to controls, suggesting a defect in excess Cu handling mechanisms (Fig. 1D). Higher Cu concentrations were equally toxic to control and ATP7A^M1311V^ fibroblasts.

To investigate copper uptake and cellular accumulation, we employed the ICAGEN XRpro® technology using X-ray fluorescence (XRF) to measure intracellular Cu levels (see Methods for details). With a short-term exposure of 250 μM copper (non-toxic for duration of experiment), ATP7A^M1311V^ fibroblasts showed significantly increased uptake and accumulation of intracellular Cu in comparison to controls with a plateau at 1 h of exposure (Fig. 1E). Replicating this experiment consistently showed 1.5 fold higher intracellular copper levels in ATP7A^M1311V^ cells compared to controls (data not shown), further highlighting that this ATP7A mutation affects Cu transport and handling.

### 2.2. Mislocalization of ATP7A^M1311V^ and impaired copper response in ATP7A^M1311V^ patient fibroblasts

**Fig. 2.**
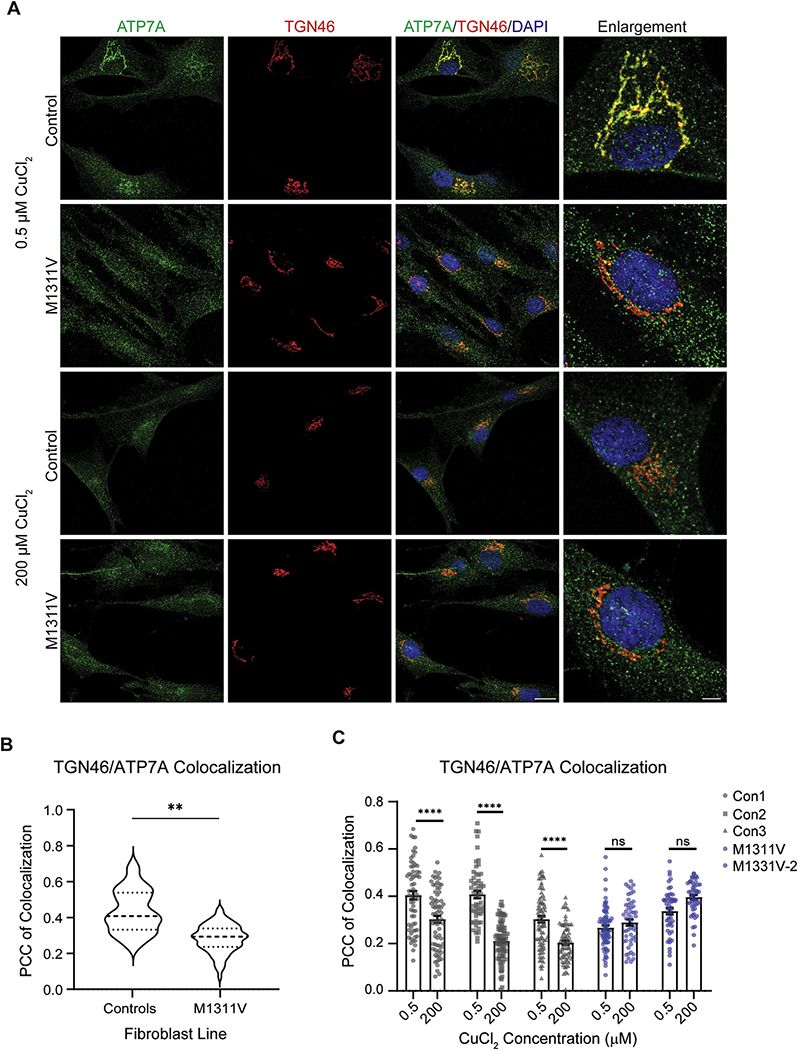
A) Immunofluorescence staining of three control fibroblast lines, as well as the ATP7A^M1311V^ cells. All fibroblasts were treated with either 0.5 μM CuCl_2_ (basal copper) or 200 μM for 2 h, then fixed and stained with antibodies against ATP7A, the Golgi marker TGN46 as well as DAPI and imaged on a confocal microscope. Shown are representative images from one control line and the ATP7A^M1311V^. The scale bars denote 20 μm and 5 μm for the enlarged images. B) Pearson’s correlation coefficient (PCC) of colocalization was computed for the various fibroblast lines under basal copper conditions, comparing the average of the three control fibroblast lines and the ATP7A^M1311V^ fibroblasts. 20–35 cells were measured per line (control is the average of three control lines). C) PCC of colocalization was used to measure colocalization of ATP7A and TGN46 under basal and 200 μM copper conditions. Asterisks denote significance within each cell line, comparing the treated and untreated conditions (*p < 0.05, **p < 0.01, ***p < 0.001, ****p < 0.0001). 50–100 cells were measured per line per condition.

Disease-associated variants of ATP7A which do not result in loss of protein are frequently associated with a mislocalization of ATP7A, as well as occasional impaired response to copper exposure (Skjorringe et al., 2017). We examined the subcellular localization of ATP7A^M1311V^ under basal copper conditions in ATP7A^M1311^ fibroblasts compared to three control fibroblast lines. ATP7A is predominately located at the Golgi apparatus, facilitating copper loading to cuproenzymes and relocates to the plasma membrane to facilitate copper efflux when intracellular copper levels increase (Kaler, 2011). We used TGN46 as a TGN marker (Fig. 2A and B), and the Pearson’s coefficient of correlation between ATP7A and TGN46 as a measure of ATP7A co-localization at the TGN. The ATP7A^M1311V^ mutation resulted in a more diffuse mutant protein staining throughout the cell (Fig. 2A top right panels) and significant reduction in ATP7A protein at the TGN under basal (0.5 μM) condition (Fig. 2B). Treatment of control fibroblasts with 200 μM copper for 2 h resulted in ATP7A translocation out of the TGN and reduced correlation of ATP7A with TGN46 (Fig. 2A and C). The ATP7A^M1311V^ fibroblasts showed no response to 200 μM copper treatment, with no changes in co-localization with TGN46 as shown by no change in the Pearson’s correlation coefficient (Fig. 2A and C).

To further monitor ATP7A translocation to the plasma membrane, we performed cell surface biotinylation assays. Fibroblast lines exhibited little plasma membrane- associated ATP7A (Fig. S1D), so we used another cell line often used for surface biotinylation studies, HeLa, expressing either wild type or ATP7A^M1311V^. In cells expressing wild type ATP7A, increased protein was detected at the cell membrane upon treatment with copper, indicating translocation to the plasma membrane (Fig. S1C). In contrast, ATP7A^M1311V^ expressing cells exhibited lower basal levels of ATP7A^M1311V^ at the surface and reduced translocation of ATP7A^M1311V^ in response to CuCl_2_ (Fig. S1C).

### 2.3. Altered expression of copper metabolism proteins and mislocalization of ATP7A in ATP7A^M1311V^ patient iPSC-MNs

**Fig. 3.**
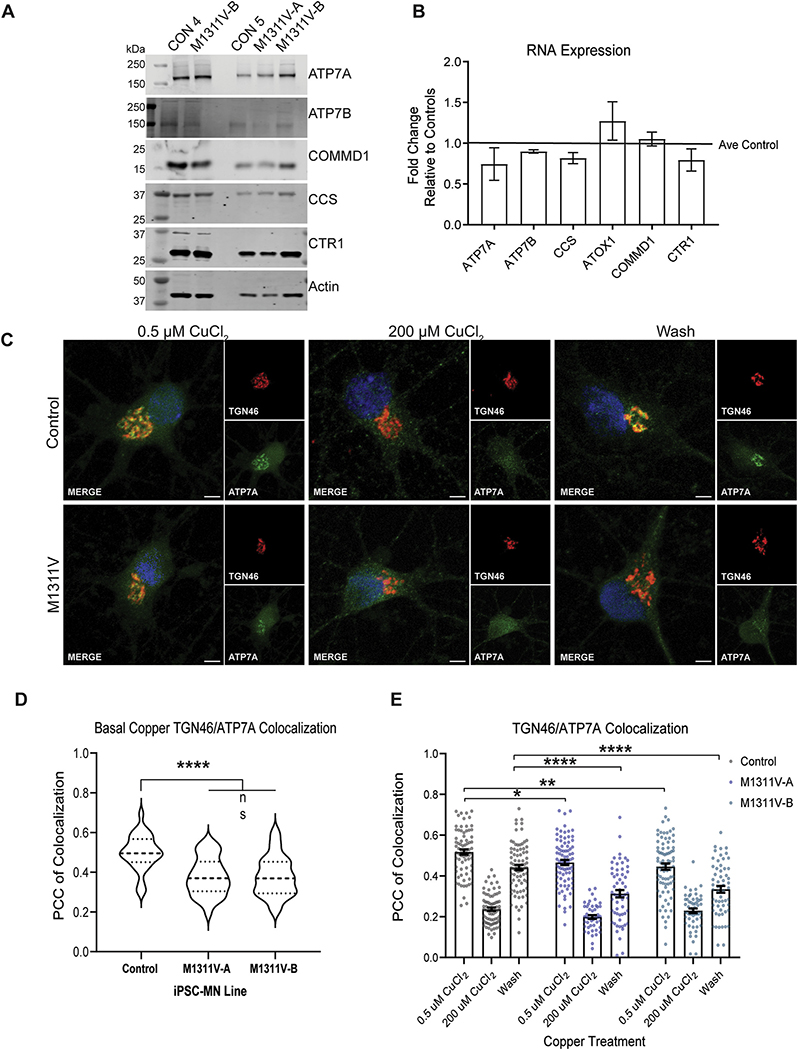
A) Control and two different clones of ATP7A^M1311V^ iPSCs (A and B) were differentiated into motor neurons, and cells were lysed for total protein extraction at days 58–66 of differentiation. Two independent differentiations were performed and western blot for the various indicated proteins were performed. B) RNA was prepared from two independent differentiations of control and ATP7A^M1311V^ iPSC-MN as in A) and mRNA levels of the indicated genes were measured, normalized to GAPDH control, and plotted as fold increase of M1311V iPSC-MNs over control MNs. C) Control and ATP7A^M1311V^ iPSC-MN monocultures were prepared and localization of ATP7A, TGN46, and DAPI was observed by immunohistochemistry at day 60 of differentiation. Neurons were identified by MAP2 positive staining (not shown). Shown are representative images from control and one ATP7A^M1311V^ iPSC-MN clone, post-treatment with basal copper (0.5 μM) for 3 h, 200 μM CuCl_2_ for 3 h, or the latter, followed by copper washout for 3 h. Scale bars denote 15 μm. D) PCC of colocalization of ATP7A and TGN46 at basal copper conditions was calculated for 50–80 cells from 1 control line and 2 clones of the same ATP7A^M1311V^ iPSC-MN line, cultured on mouse astrocytes. Asterisks denote significance (*p < 0.05, **p < 0.01, *** = p < 0.001, **** = p < 0.0001). E) PCC of colocalization was computed similar to D) for iPSC-MNs cultured without astrocytes, under basal copper conditions (0.5 μM), treated with 200 μM CuCl_2_ for 3 h, or treated with 200 μM CuCl_2_ for 3 h followed by copper washout for 3 h. 30–35 cells from the same control line and two ATP7A^M1311V^ clones at day 57 of differentiation were measured. Asterisks denote significance (*p < 0.05, **p < 0.01, ***p < 0.001, ****p < 0.0001).

We next determined if ATP7A^M1311V^ mislocalization occurs in motor neurons. First, we reprogrammed ATP7A^M1311V^ patient-derived fibroblasts and generated three clonal iPSC lines that were subsequently differentiated into motor neurons. After generation of iPSC-MNs we measured protein and mRNA levels of copper metabolism proteins (Fig. 3A and B). ATP7A protein expression was significantly increased in ATP7A^M1311V^ iPSC-MNs when compared to healthy controls, while ATP7B expression was reduced (Figs. 3A and S2A). No significant changes in RNA levels of either transporter or copper binding proteins were observed (Fig. 3B).

Similar to ATP7A^M1311V^ fibroblasts, there was less M1311V ATP7A protein at the TGN under basal conditions compared to controls, as evidenced by significantly reduced co-localization with TGN46 in neurons co-cultured on astrocytes (Fig. 3D) and without astrocytes (Fig. 3C and E). When iPSC-MNs were treated with CuCl_2_, less ATP7A was found at the TGN in control iPSC-MNs, suggesting a translocation from the Golgi to the plasma membrane. ATP7A^M1311V^ iPSC-MNs did respond to copper with similar decreased ATP7A protein at the Golgi (Fig. 3C and E), as well as similar localization to the cell surface by biotinylation assays (Fig. S2B). Interestingly, while a copper washout step was able to recover ATP7A localization back to the Golgi in control iPSC-MNs, ATP7A^M1311V^ iPSC-MNs failed to relocate back to the TGN (Fig. 3C and E). These findings highlight a defect in ATP7A^M1311V^ targeting or retention of the mutant protein to the Golgi apparatus.

### 2.4. Morphological and functional deficits in ATP7A^M1311V^ iPSC-MNs

**Fig. 4.**
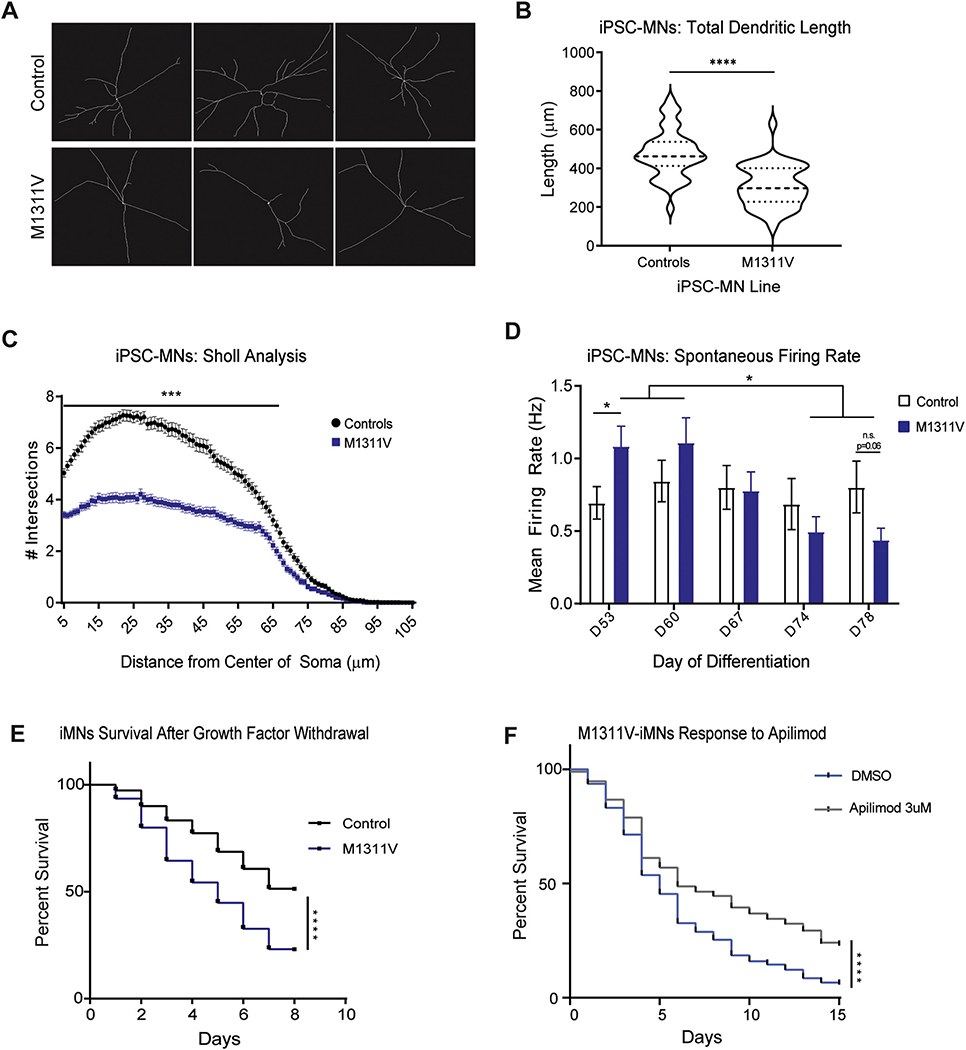
A) Control and ATP7A^M1311V^ patient-derived iPSC-MN were differentiated for 64 days and immunostaining for MAP2 was performed to trace neuronal processes. Shown are three examples from each genotype. B) Neuronal tracing was performed as described in A) and total dendritic length was calculated for 20 cells for 2 control lines and 20 cells for 2 clones of the same ATP7A^M1311V^ line, each with 2 differentiations. Asterisks denote significance (*p < 0.05, **p < 0.01, ***p < 0.001, ****p < 0.0001). C) Sholl analysis of iPSC-MNs was performed for 20 cells from each of 2 control lines and 20 cells for each of 2 clones of the same ATP7AM1311V line, with 2 differentiations each for a total of 80 cells per group. Asterisks denote significance (*p < 0.05, **p < 0.01, ***p < 0.001, ****p < 0.0001). D) Mean firing rate (spikes/s) of 2 controls and 2 clones of M1311V iPSC-MNs recorded over 25 days (*n* = 12 wells per line, 2 differentiations). E) Induced-motor neurons (iMN) were generated from control and ATP7A^M1311V^ iPSCs, transduced with HB9::RFP reporter and plated on cortical glial cells in 96-well plates. Growth factors were withdrawn at day 17, and longitudinal tracking of RFP in neurons was initiated. Neurons were scored as dead when their soma was no longer detectable by RFP fluorescence. For each line, the survival data from 50 iMNs were selected randomly, and used to generate the survival curve. Equal numbers of neurons from two individual replicates were used and two independent repeats of the experiment were performed. Statistical significance was evaluated by comparing test and controls by log-rank test. Asterisks denote significance (*p < 0.05, **p < 0.01, ***p < 0.001, ****p < 0.0001). F) iMNs were generated as in E from ATP7A^M1311V^ iPSCs, and survival under growth factor withdrawal conditions was measured for cells treated with vehicle (DMSO) or 3 μM Apilimod. iMNs quantified from 3 biologically independent iMN conversions per condition, with each condition run in triplicates. Asterisks denote significance (p < 0.0001). Hazard ratios were calculated to be 0.61.

We next asked whether ATP7A^M1311V^ expressing neurons exhibited motor neuron phenotypes associated with ALS. To test this hypothesis, we monitored ATP7A^M1311V^ iPSC-MNs for alterations in cell morphology and electrophysiological properties. Neuronal processes and dendrites were traced in differentiated iPSC-MNs using Microtubule associated protein 2 (MAP2) immunostaining (Fig. 4A). Compared to controls, ATP7A^M1311V^ iPSC-MNs have reduced overall dendritic length (Fig. 4B) and reduced dendritic complexity as measured by Sholl analysis (Fig. 4C).

The fundamental measure of neuronal function is the ability to generate electric potentials. Using a multielectrode array (MEA) platform to record neuronal activity, we measured longitudinal spontaneous electrical firing in control and ATP7A^M1311V^ iPSC-MNs. At early time points following differentiation, ATP7A^M1311V^ iPSC-MNs exhibited hyperexcitability when compared to control iPSC-MNs, then transitioned to hypoexcitability at later time points (Fig. 4D). Such a phenotype and transition in neuronal excitability is remarkably similar to previously described motor neuron models of familial ALS, notably in iPSC-MNs carrying TDP-43 or C9orf72 disease causing mutations (Devlin et al., 2015).

We also investigated the effect of the ATP7A^M1311V^ mutant protein on motor neuron viability. We directly induced motor neurons from patient derived fibroblasts (iMNs) using previously published protocols (Shi et al., 2018). When compared to control iMNs, survival of ATP7A^M1311V^ iMNs was significantly reduced upon withdrawal of neurotrophic factors (Fig. 4E) suggesting deleterious effects of the mutation on neuronal survival.

Given the defects in cellular trafficking associated with ATP7A^M1311V^, we asked whether the PIKFYVE inhibitor, Apilimod, which rescues *C9orf72* expansion-associated vesicular trafficking defects and iMNs degeneration, could have a similar effect on ATP7A^M1311V^ neurons. PIKFYVE inhibition increases autophagosome-lysosome fusion (Martin et al., 2013), and has recently been shown to reverse phenotypic changes associated with C9orf72 haploinsufficiency, increasing cellular survival under growth factor withdrawal or excess glutamate conditions (Shi et al., 2018). We tested Apilimod on ATP7A^M1311V^ iMNs and observed a maximum dose response at 2–3 μM (Fig. S3A), and used 3 μM for all subsequent assays. Similar to C9orf72 iMNs, Apilimod treatment of ATP7A^M1311V^ iMNs significantly improved survival under neurotrophic withdrawal conditions when compared to vehicle-control treated neurons (Fig. 4F).

Taken together, our findings demonstrate altered morphology, function and susceptibility to stressors of iPSC-MNs carrying the ATP7A^M1311V^ mutation, highlighting the potentially deleterious effects of this mutation on motor neurons.

### 2.5. Expression of ATP7A^M1311V^ in Drosophila impairs locomotor function

**Fig. 5.**
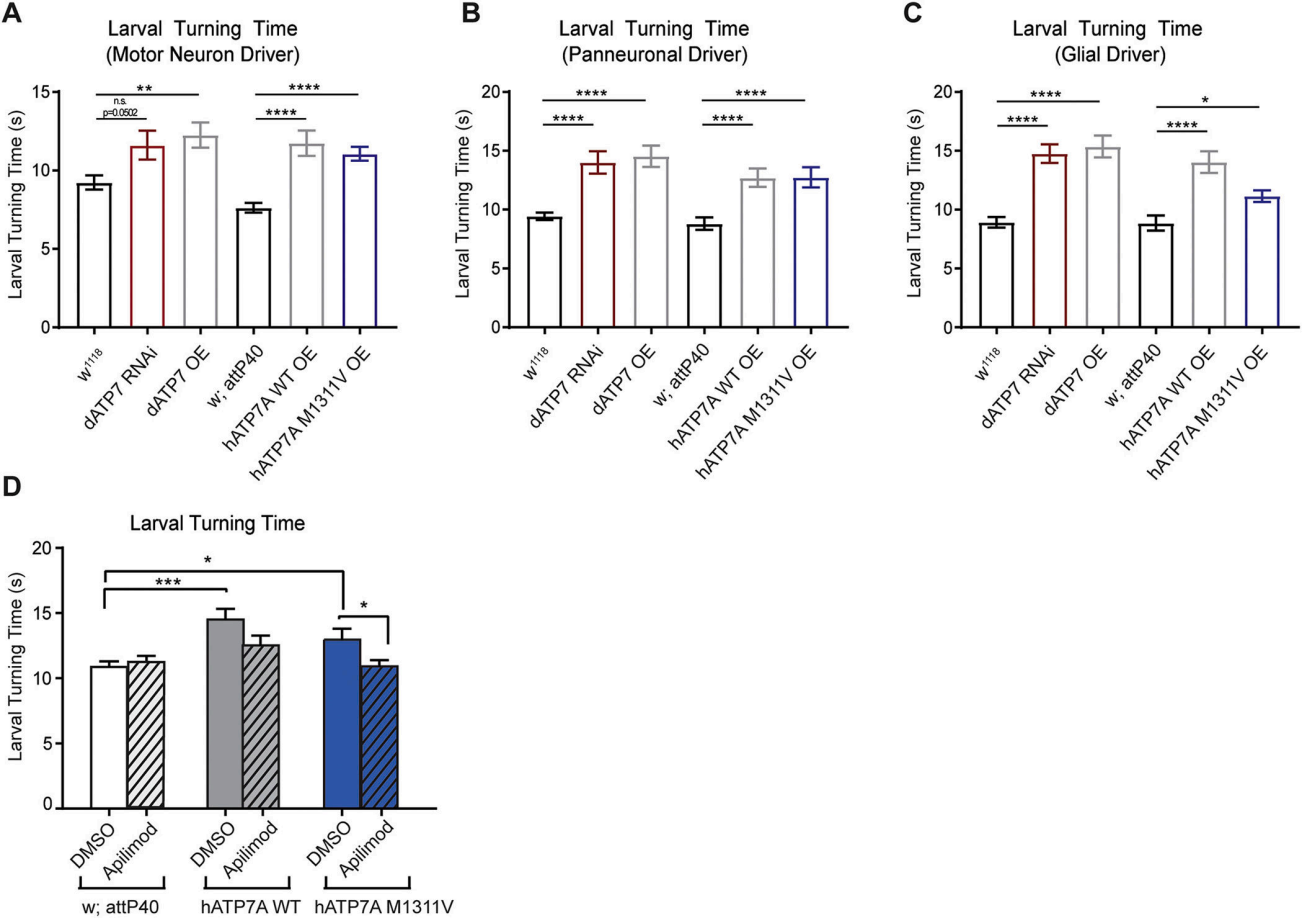
The M1311V ATP7A variant causes locomotor dysfunction that phenocopies copper homeostasis deficits in Drosophila. A) dATP7 RNAi knock-down or overexpression (OE) as well as hATP7A WT or M1311V overexpression (OE) in motor neurons using D42 GAL4 causes increased larval turning times compared to genetic background controls (w^1118^ for dATP7 and w; attP40 for hATP7A). B) dATP7 RNAi knock-down or overexpression (OE) as well as hATP7A WT or M1311V pan-neuronal overexpression (OE) using elav GAL4 causes increased larval turning times compared to genetic background controls (w^1118^ for dATP7 and w; attP40 for hATP7A). C) dATP7 RNAi knock-down or overexpression (OE) as well as hATP7A WT or M1311V overexpression (OE) in all glia using repo GAL4 causes increased larval turning times compared to genetic background controls (w^1118^ for dATP7 and w; attP40 for hATP7A). Statistical significance was determined using a Kruskal-Wallis One Way ANOVA. Asterisks denote significance (*p < 0.05, **p < 0.01, ***p < 0.001, ****p < 0.0001). D) Larval turning assays were performed similar to Fig. 6A) in larvae expressing hATP7A WT or M1311V in motor neurons and fed 15 μM Apilimod or DMSO.

To further probe the effect of the ATP7A^M1311V^ variant on motor neuron function in vivo we used *Drosophila* as a model. The *Drosophila* genome contains a single orthologue, dATP7, which was previously shown to be an important modulator of copper efflux (Balamurugan et al., 2007). RNAi knockdown or overexpression of dATP7 specifically in motor neurons using the GAL4-UAS system (Brand and Perrimon, 1993) induced impaired locomotor function, as indicated by larval turning assays (Fig. 5A). These experiments show that dATP7 dosage is critical to normal locomotor function, as either loss or gain of dATP7 causes a significantly increased larval turning time compared to w^1118^ genetic background controls. Further substantiating these observations are findings that dATP7 knock-down or overexpression in all neurons (with Elav GAL4) or in all glial cells (with Repo GAL4) cause similar larval turning defects suggesting that copper homeostasis is important for proper motor function in Drosophila (Fig. 5B and C). These locomotor function deficits were not associated with morphological changes at the larval neuromuscular junctions (data not shown). To determine the in vivo effects of the ATP7A^M1311V^ variant on locomotor function, we generated *Drosophila* expressing human wild type ATP7A (hATP7A) or ATP7A^M1311V^ under the control of the UAS promoter (see Materials and Methods). Surprisingly, overexpression of either wildtype or M1311V hATP7A in the developing eye neuroepithelium did not cause a visible phenotype when compared to w;attP40 genetic background controls (data not shown). In contrast, overexpression of hATP7A (wild-type or M1311V) in neurons or glia causes significant locomotor function deficits when compared to w;attP40 genetic background controls, as indicated by larval turning assays (Fig. 5A, B and C). Since the expression levels of wild-type and mutant ATP7A are comparable (Fig. S3B), these results suggest that perturbations in ATP7A dosage and function are responsible for motor deficits, consistent with the overexpression and loss of the Drosophila ortholog (Fig. 5A, B and C). Taken together, these findings suggest that glia and motor neurons but not photoreceptor neurons are susceptible to alterations in copper homeostasis.

To address the contribution of vesicular ATP7A trafficking at the PIKFYVE/Rab5 step to this in vivo locomotor deficit, we used a similar pharmacological approach as in our in vitro iMN cell culture model. Fly larvae were fed 15 μM Apilimod and tested for locomotor function. Interestingly, Apilimod treatment of larvae overexpressing hATP7A M1311V but not wild-type hATP7A caused a significant improvement in larval turning time compared to vehicle control-fed larvae (Fig. 5D). These experiments suggest that PIKFYVE inhibition via Apilimod protects mutant hATP7A dependent locomotor defects in vivo, which is consistent with the results in patient derived iMNs.

## 3. Discussion

An ALS patient with brachial amyotrophic diplegia/flail arm syndrome was found to carry a genetic variant of ATP7A, ATP7A^M1311V^. To better understand the potential contribution of this mutant ATP7A to neuronal dysfunction, we performed a functional analysis of patient-derived fibroblasts, as well as patient-derived iPSC-MNs, and compared them to control cell lines. We detected reduced levels of ATP7A^M1311V^ protein in the TGN in both patient derived fibroblasts and iPSC-MNs when compared to cells expressing wild type ATP7A, as well as reduced translocation of ATP7A^M1311V^ from the TGN in response to excess copper treatment in fibroblasts. In addition, iPSC-MN-ATP7A^M1311V^ showed a defect in relocating ATP7A back to the TGN following copper washout highlighting a defect in adequate targeting of the protein to Golgi/response to copper exposure. This manifested in enhanced copper uptake and reduced survival of fibroblasts expressing ATP7A^M1311V^. Since ATP7A functions at the TGN to load copper onto a variety of cuproenzymes, we propose that its reduced presence at the Golgi may result in deleterious cellular functions. The fact that less ATP7A^M1311V^ was found at the TGN under basal conditions, and that we could not detect increased levels of the protein at the plasma membrane, yet total ATP7A^M1311V^ protein levels are increased in iPSC-MNs expressing the M1311V variant, suggests that the mutant copper transporter is trapped in trafficking vesicles. In fact, modulation of vesicular trafficking using the PIKFYVE inhibitor Apilimod rescued ATP7A^M1311V^ iMNs survival under neurotrophic factor withdrawal conditions. Apilimod was similarly able to rescue larval turning time in *Drosophila* expressing human ATP7A^M1311V^, further highlighting a vesicular defect associated with the M1311V mutation. Li et al. reported that the ATP7A^P1386S^ distal motor neuropathy-associated mutation aberrantly localized mutant protein to axons under basal copper conditions potentially due to impaired interaction with AP-1 and/or AP-2 in endosomal vesicles, a phenotype associated with altered calcium signaling patterns after glutamate stimulation (Yi and Kaler, 2015). Since the AP-1 adaptor typically sorts cargo shuttling between endosomes and TGN (Robinson, 2004), while AP-2 associates with cargoes shuttling back from the plasma membrane, similar impaired interactions of ATP7A^M1311V^ with AP-1/2 may be responsible for impaired cellular localization of the transporter under basal copper conditions and will be examined in future studies. Since impaired TGN targeting of ATP7AM1311V was observed at basal levels and in response to copper washout, but not copper exposure, one can suggest that the transport defect is at the level of the forward trafficking to the TGN as opposed to exit from the Golgi, although more investigations are needed to fully dissect that phenotype.

ATP7A^M1311V^ iPSC-MNs exhibit altered morphology characterized by decreased total dendritic length, less complex neuronal branching, and abnormal neuronal firing. Recently, ATP7A^M1311V^ was associated with glutamate vulnerability and reduced firing action potential (Yun et al., 2020), with both of these phenotypes rescued when the mutation was corrected with CRISPR gene editing technologies. Elevated copper at synapses has been recently imaged in response to calcium in neurons using fluorescent copper sensors CS3 (Dodani et al., 2011); it can reach up to 100–250 μM in synapses (Kardos et al., 1989) where it can bind and modulate the function of the *N*-methyl-d-aspartate (NMDA), α-amino-3-hydroxy-5-methyl-4-isoxazolepropionic acid (AMPA), and Gamma-Aminobutyric Acid (GABA) receptors, as well as voltage-gated Ca channels (Gaier et al., 2013). Copper exposure can have biphasic effects on rat hippocampal neurons, with acute exposure blocking synaptic transmission, while longer treatments enhance AMPA receptor-mediated currents and increase glutamate ionotropic receptor AMPA type subunit 1 (GluA1) and postsynaptic density protein 95 (PSD-95) levels (Peters et al., 2011), highlighting the complex effects of copper on synaptic function. It is thus not surprising that we found ATP7A^M1311V^ motor neurons with impaired copper handling to show uncharacteristic excitability phenotypes. ATP7A^M1311V^ iPSC-MNs also displayed significantly increased cell death in response to neurotrophic factor deprivation when compared to iPSC-MNs expressing wild type ATP7A.

ATP7A^M1311V^ overexpression in *Drosophila* was associated with defects in larval turning, similarly to the overexpression of the wild type version of ATP7A, regardless of whether the copper transporter was expressed in motor neurons or glia. Interestingly, expression in the developing neuroepithelium produced no obvious phenotypes highlighting the importance of copper homeostasis in motor neurons. The fact that under- or over-expression of dATP7 and over-expression of either wildtype or M1311V hATP7A all induced similar locomotor deficits reminiscent of TDP-43 based models of ALS (Estes et al., 2011) in *Drosophila* larvae highlights the important role that precise regulation of ATP7A expression and, consequentially, copper homeostasis, plays in maintaining motor function. Recently, loss of function of ATP7A in *C. elegans* resulted in defects in motility, decline in motility over age as well as body wall muscle defects (Soh et al., 2020). This is notable given the upregulation of ATP7A expression observed in patient-derived iPSC-MNs, which may be sufficient to impair motor neuron function independent of our evidence for altered ATP7A function due to the M1311V amino acid substitution. Given that whole brain copper levels rise between 2 and 3.5 fold between infancy and adulthood in rats (Tarohda et al., 2004) coincident with sharp increases in ATP7A expression in ependymal cells (Niciu et al., 2006), the effect of this ATP7A mutation on adult *Drosophila* locomotion and neuromuscular function will be investigated in the future.

In recent years, the number of known genes linked to familial forms of ALS or risk factors for ALS has risen considerably (Chia et al., 2018; Renton et al., 2014); however, the factors responsible for the vast majority of ALS cases remain unidentified. While environmental exposures may contribute to sporadic forms of ALS, it is likely that subtle genetic or epigenetic alterations which are not independently causal combine with each other and environmental factors to induce sporadic disease. The frequency of the ATP7A^M1311V^ variant within the general population argues against this mutation as directly causal for ALS, yet ATP7A^M1311V^ iPSC-MNs exhibit phenotypes remarkably similar to other models of ALS. Namely, the defects in synaptic complexity of neurons, as well as aberrant excitability, with both the early hyperexcitation and the later hypoexcitation phenotypes are all hallmarks of C9orf72-associated ALS (Starr and Sattler, 2018).

Taken together, we have shown that the ATP7A^M1311V^ variant alters ATP7A protein subcellular distribution and function, as well as its translocation in response to copper stimulation. Patient-derived motor neurons harboring this variant show altered excitability, and increased vulnerability to growth factor withdrawal, motor neuron phenotypes associated with ALS and neurodegeneration. The role of copper homeostasis in ALS remains unclear, though significant increased levels of copper as well as the other metal ions have been reported in ventral areas of spinal cords from sporadic ALS cases (Kurlander and Patten, 1979), but later reports have failed to show clear indications of abnormalities in copper handling proteins levels and functions in human sporadic ALS (Tokuda and Furukawa, 2016). Recently, a copper donor routinely used for PET imaging, Cu-ATSM induced dramatic survival improvement in the transgenic SOD1^G93A^ mouse model of ALS (Vieira et al., 2017; Williams et al., 2016) and is currently in early phase clinical trials for ALS (NCT04082832). This development, combined with recent descriptions of mutant ATP7A-induced distal motor neuropathies and our current observations regarding ATP7A^M1311V^, supports a focus on copper regulation as a key mechanism in progressive motor neuron degeneration.

## 4. Materials & methods

### 4.1. Fibroblast and generation of patient derived iPSC lines

Induced pluripotent stem cells (iPSCs) were generated as previously described (Holler et al., 2016). In brief, early passage fibroblast (<P10) were grown to approximately 50–80% confluency in fibroblast medium consisting of 10% ES-qualified FBS (Life Technologies, 0.1 mM NEAA, 55 μM β-mercaptoethanol, high glucose DMEM (Life Technologies). On Day 0, fibroblasts were transduced with the three Sendai viruses (KOS, hc-Myc, hKlf4, each at an MOI of 5). Cells were fed every other day for 7 days with fibroblast medium. On day 7, cells were passaged onto vitronectin (Life Technologies) coated dishes at a density of 250,000 to 500,000 cells/well. Beginning on day 8, cells were fed every day in Essential 8 medium (Life Technologies). iPSC colonies were manually picked and transferred to vitronectin coated dishes; after 2 passages, iPSCs were transferred to Matrigel coated dishes. iPSCs were maintained on Matrigel coated dishes and mTesR1 medium (Stem Cell Technologies). iPSCs were passaged every 5–7 days using ReLeSR (Stem Cell Technologies). Cells were checked for pluripotency markers by immunofluorescence (Fig. S2C) using the following markers: Octamer-binding transcription factor 4 (Oct4; 1:400; Cell Signaling Technologies); Stage-Specific Embryonic Antigen 4 (SSEA; 1:500; Cell Signaling Technologies); Nanog homeobox (Nanog; 1:200; R&D Systems); Sex Determining Region Y-Box 2 (Sox2;1:200: ThermoScientific); Tra1–60 and Tra1–81 (1:150 each; Millipore). Correct chromosomal numbers were checked by karyotyping at WiCell (Madison, Wisconsin, USA; Fig. S2D).

### 4.2. Glutathione cellular stress assay

Assay of glutathione content was performed on cells using the luminescence-based GSH/GSSG-Glo Assay from Promega as suggested by the manufacturer. The kit allows the measurement of both oxidized and total glutathione levels in order to compute the ratios of reduced to oxidized glutathione. Briefly the cells are lysed in total glutathione or oxidized glutathione lysis buffers directly in the culture wells following treatments, followed by addition of luciferin. Luminescence is then read and background readouts are subtracted from all measurements. 5 wells per experimental group were used.

### 4.3. iMN and iPSC-MN culture

For all experiments other than the growth factor withdrawal survival assay, patient derived iPSCs were cultured and differentiated as previously described (Moore et al., 2019; Zhang et al., 2015). In brief, patient and healthy individual-derived iPSCs were grown in mTeSR media (Stem Cell Technologies) and manually cleaned to remove spontaneous differentiation. Karyotyping and analysis of pluripotency markers was performed as a quality control for the cells (Fig. S2C and D). After two passages, the iPSC differentiation process was initiated. After 40 days of differentiation, iPSCs were counted and plated on prepared plates for experiments, which were conducted between day 55 and 65. Cells were plated on mouse primary astrocytes for morphological and electrical activity analyses; on coverslips coated with 0.07% branched polyethylenimine (PEI) (Sigma #408727) and 3.3 μg/ml laminin (Thermo #23017–015) for copper treatment and colocalization assays; or maintained in 1:100 Matrigel coated flasks for protein and RNA analyses.

The induced-motor neurons (iMN) were generated for survival assays. iPSC-derived secondary fibroblasts were seeded at least 48 h before viral transduction for each experiment in 96-well plates that were sequentially coated with gelatin (0.1%, 1 h at 37 °C) and laminin (overnight at 4 °C). Seven iMN transcription factor-encoding retroviruses were added in fibroblast medium with 5 μg/ml polybrene for 24 h followed by transduction of lentivirus encoding Hb9::RFP reporter. On day 5, primary mouse cortical glial cells from P2-P4 ICR pups (male and female) were added to the transduced cultures in glia medium containing MEM (Life Technologies), 10% donor equine serum (HyClone), 20% glucose (Sigma-Aldrich), and 1% Pen-Strep. On day 6, cultures were switched to N3 medium containing DMEM/F12 (Life Technologies), 2% FBS, 1% penicillin/streptomycin, N2 and B27 supplements (Life Technologies), 7.5 μM RepSox (Selleck), and 10 ng/ml each of GDNF, BDNF, FGF, and CNTF (R&D).

### 4.4. Mouse primary astrocyte culture

Astrocytes cultures were prepared from post-natal day 0–3 mouse pups. Six to eight whole cortices were dissected using cold 1× Hanks’ Balanced Salt Solution (HBSS) no calcium, no magnesium (Gibco # 14185–022) supplemented with 30 mM glucose, 10 mM Hepes, 1 mM Sodium Pyruvate and 1% Pen-Strep. The cortices were then digested using 0.1 mg/ml DNase (Sigma) and 1.5 mg/ml Papain (Worthington) in dissection buffer and incubated for 15 min at 37 °C water bath. The cortices were then washed and resuspended in astrocyte media (DMEM, 10% fetal bovine serum and 1% pen/strep). Cell suspension was then pass through a cell strainer (40 μm - Falcon). Astrocytes were plated into T75 flasks (1 cortex/flask) until cells reach confluency. Astrocytes cultures were used within 10 culture days. Cells were then counted and seeded at a desirable density on 1.5 mg/ml collagen (PureCole Advanced Biometrix) coated coverslips on 24 well plates (250000–300,000 cells/well) or MEA 48 well plates (55,000 cells/well). The astrocytes were ready to use 3–4 days after plated when a confluent monolayer was formed.

### 4.5. Biotinylation and western blots

Venus-ATP7a-WT and P1386S mutants were kindly provided by S. Kaler (NIH). QuikChange XL mutagenesis (Stratagene) was used to generate the M1311V mutant from the Venus-ATP7a-WT backbone by PCR. Constructs were grown in Copycutter EPI400 *E*-coli bacteria (Epicentre), DNA was isolated and transfected into Hela cells (grown in DMEM supplemented with 10% FBS) using Lipofectamine 2000 according to manufacturer’s recommendations (Thermo Scientific). Cells were treated with 200 μM CuCl_2_ for 2 h, washed twice with ice-cold phosphate buffered solution (PBS) and incubated with 1 mg/ml EZ-link NHS-SS-Biotin (Thermo Scientific, #21331) for 30 min at 4 °C, shaking. Cells were then quenched with 10 mM Glycine in PBS for 20 min and scraped on ice. After pelleting, cell lysis was performed in radioimmunoprecipitation (RIPA) lysis buffer (150 mM NaCl, 50 mM Tris-HCl pH 7.4, 5 mM EGTA, 1% TritonX-100 supplemented with protease inhibitor cocktail (Sigma)) with additional 0.5% sodium deoxycholate and 0.1% sodium dodecyl sulfate (SDS). Lysates were freeze-thawed in liquid nitrogen and spun down at 6500 *g* for 10 min. Protein quantification was performed and 10% of the lysates were stored to be used as input, while the remaining proteins were incubated with Neutravidin Agarose Resin beads (Thermo Scientific), washed in lysis buffer. The protein-bead mixture was incubated overnight at 4 °C. Following the immunoprecipitation, the beads were washed four times with RIPA, and once with 50 mM Tris-HCl, before eluting in lithium dodecyl sulfate (LDS) elution buffer. The protein-bead complexes were then boiled at 80 °C for 10 min., and spun down at 10,000 *g* for 1 min. The supernatants were loaded on 3–8% Tri-acetate gels (Thermo Scientific) and ran for 1 h at 100 V before transferring onto Immobilon FL (Millipore) PVDF membranes. Membranes were subsequently blocked in Odyssey blocking buffer and probed with primary and secondary antibodies. Signals were imaged using the Odyssey CLx Imager (LiCor), and densitometric analysis was performed using the ImageStudio 4.0 software from LiCor. For western blots on iPSC-MNs and fibroblasts, cells were scraped and lysed in RIPA supplemented with protease and phosphatase inhibitor cocktails (Calbiochem). Proteins were separated on Nupage Bis-Tris 4–12% protein gels from ThermoFisher Scientific and transferred onto PVDF membranes. Primary antibodies used were: rabbit polyclonal Copper Chaperone For Superoxide Dismutase (CCS), Copper Metabolism Domain Containing 1 (COMMD1) (Proteintech #22802, and #11938), rabbit monoclonal ATOX1, ATP7b, and Copper Transporter 1 (CTR1) (Abcam ab154179, ab131208, and ab 129,067 respectively), mouse monoclonal ATP7A (Santa Cruz sc376467). Mouse anti-Vinculin and anti-Actin loading controls were from Abcam (ab18088) and Millipore (MAB1501), respectively.

### 4.6. Gene expression of copper binding proteins and transporters

RNA was isolated from cell pellets using the RNeasy Mini Kit (Qiagen) and quantified using Nanodrop. 500 ng of RNA were DNAse treated and reverse transcribed using the RT^2^ First Strand Synthesis Kit (Qiagen). TaqMan Fast Advanced Master Mix and TaqMan Gene Expression Assays (Applied Biosystems) were used to quantify gene expression normalized to GAPDH expression. qRT-PCRs were run according the TaqMan Fast Advanced Master Mix manufacturer’s protocol in a 96-well format on a QuantStudio 6 Flex system (Applied Biosystems). Log_2_ fold changes were calculated using the delta-delta Ct method. TaqMan Gene Expression Assays used: ATP7A – HS_00163707 m1, ATP7B – HS_00163739 m1, CCS – HS_00192851 m1, ATOX1 – HS_00187841, COMMD1 – HS_00415059, SLC31A1 – HS_00977266 g1, GAPDH – HS_99999905 m1 (Applied Biosystems).

### 4.7. MTT viability assay

Fibroblasts were isolated from skin biopsies of ALS patients and healthy controls and cultured in media consisting of DMEM (Gibco) with 10% Fetal Bovine Serum and 1% Pen-Strep. For viability assays, fibroblasts were seeded in a 96-well plate at a density of 3000 cells/well in DMEM +10% FBS+ 100 U/ml penicillin, 100 μg/ml streptomycin and incubated overnight. On day 2, cells were treated with serial dilutions of CuCl_2_. After incubation at 37 °C for 24 h, 20 μl of 5 mg/ml (3-(4,5-Dimethylthiazol-2-yl)-2,5-Diphenyltetrazolium Bromide) (MTT) (Life Technologies M6496) were added to each well, and the plates were incubated for an additional 3 h at 37 °C. MTT was aspirated, 150 μl DMSO was added and cells were agitated on an orbital shaker for 15 min. The absorbance was read at 595 nm using a microplate reader. Each condition was carried out in quadruplicate, background absorbance was subtracted, and MTT absorbance was calculated compared to control cells. Results from both control lines were averaged and pooled and the absorbance of untreated cells set to 100%. Two-way ANOVA was used to compare the two groups.

### 4.8. Immunofluorescence microscopy (fibroblasts and iPSCs)

Fibroblasts were plated on uncoated coverslips in a 24-well plate. IPSC-MNs were plated on coverslips in a 24-well plate either on top of primary murine astrocytes on collagen-coated coverslips (morphological analyses) or without astrocytes on PEI:laminin coated coverslips (colocalization analysis). Cells were fixed in 4% paraformaldehyde (PFA) for 20 min and washed with PBS. Fixed cells were permeabilized in blocking solution (10% normal goat serum, 0.5% bovine serum albumin in PBS) with 0.1% triton for 20 min. Fresh blocking solution was added for 1 h, and primary antibodies diluted in blocking solution were added to incubate overnight at 4 °C. The next day, cells were washed with PBS and blocked again for 1 h. Secondary antibodies in blocking solution were incubated for 1 h at room temperature. After additional washing, coverslips were mounted on slides using ProLong Gold Anti-fade Mountant containing DAPI (Life Technologies) and imaged using a 63× oil or 40× water objective on a Zeiss 800 confocal microscope. Primary antibodies used: chicken anti-ATP7A (Abcam ab13995, 1:1000), rabbit anti-TGN46 (Novus Biologicals NBP1–49643, 1:500), and guinea pig anti-Map2 (Synaptic Systems 188,004, 1:1000). Alexa Fluor secondary antibodies were used at a dilution of 1:500 (Invitrogen).

### 4.9. Copper response experiments

Prior to being fixed and stained, fibroblasts were treated with 0.5 or 200 μM CuCl_2_ for 1 h, while iPSC-MNs were treated for 3 h with 0.5, 200 μM CuCl_2_, or the latter followed by copper washout. For the washout, cells were rinsed once with culture medium and incubated with medium containing 50 μg/ml cycloheximide (Sigma-Aldrich C7698) and 200 μM bathocuproinedisulfonic acid (BCS; Sigma-Aldrich B1125) for 3 h.

Colocalization of ATP7A with TGN46, was quantified using the Imaris 9.4 (Bitplane) image analysis software’s colocalization tool. For each cell analyzed, volumetric Surfaces were created from the TGN46 channel using a consistent threshold cutoff that excluded background signal. A colocalization channel was then created from the ATP7A and TGN46 channels within each Surface. The Pearson’s correlation coefficient of colocalization was recorded for each cell. For fibroblasts, images were taken to total at least 20 cells per line/treatment. For copper treated iPSC-MNs, at least 50 cells per line/treatment were analyzed. Basal colocalization in iPSC-MNs cocultured with astrocytes was determined in a similar manner, with at least 30 cells per line analyzed. Statistics were performed in GraphPad Prism using Tukey’s multiple comparisons test.

### 4.10. Copper uptake assay

Copper uptake and accumulation was measured in fibroblast cell lines using Icagen’s XRpro® technology that uses X-ray fluorescence (XRF) to measure metal transporter activity by quantifying chemical elements in cell populations. XRF is a well-established technology in analytical chemistry and materials science with new and developing application for biochemistry, including XRpro® (Haschke, 2014; Paunesku et al., 2006).

Fibroblasts were grown in 96-well plates precoated with poly-D lysine and fibronectin, seeded at 14,500 cells per well in DMEM with 10% FBS + 1% Glutamax and Pen-Strep. For uptake assays, culture media was removed, cells washed once with uptake buffer lacking Cu^2+^, and then incubated in 100 μl per well of uptake buffer (1.26 mM CaCl_2_, 5.33 mM KCl, 0.5 mM MgCl_2_, 0.41 mM MgSO_4_, 139 mM NaCl, 5.6 mM glucose, 10 mM HEPES, pH 7.4) + Cu^2+^ for 3 h at 37 °C with 5% CO2. Uptake reactions were stopped by removing Cu^2+^ uptake buffer and washing twice with 100 μl / well uptake buffer without Cu^2+^ that was supplemented with 1 mM trace-metal certified EDTA (BioUltra grade, Sigma #03609) and 1 mM CaCl_2_ (Sigma BioXtra, cat# 5080). Wash buffer was removed and cells were lysed overnight at 37 °C using 100 μl / well XRpro® osmotic lysis buffer containing 2.5 mM Tris-HCl pH 7.5, 1 mM EDTA, 0.25 mM Allura red AC, and 0.1 mM YCl_3_. 40 μl / well of lysate was transferred to XRpro® analysis plates, and samples were dried via vacuum centrifugation. Cu^2+^ concentrations were measured using an XRpro® X-ray fluorescence instrument equipped with a 30 W Mo target X-ray tube, a 100 μm polycapillary X-ray focusing optic a silicon drift X-ray detector. X-ray fluorescence (XRF) spectra from 1 to 20 KeV were obtained for each well. Cu signal in counts per unit time were determined by curve fitting spectra to measure area under XRF peaks. Lysate Cu^2+^ concentrations were calculated using Cu2+ titration standard curves, and normalized to relative cell number, calculated using Promega CellTiter-Glo. Four replicates were used per group.

### 4.11. iPSC morphological analyses

Control and ATP7A^M1311V^ iPSC-MNs were cultured on astrocytes, fixed, and immunostained for MAP2. Using collapsed Z stacks from confocal images of MAP2 staining, the dendrites of iPSC-MNs were traced from the center of the soma with the Imaris Filament Tracer tool (Bitplane). The total length of dendrites in a given cell was provided by the tracer. Greyscale images of traces were then imported into ImageJ and analyzed using the Sholl analysis plugin. Sholl analysis results are reported as the number of intersections between the cell’s dendrites and concentric circles drawn X pixels away from the center of the soma. Images of 20 cells per line were collected per differentiation. Two differentiations were combined and analyzed for a total of 80 cells per group. Statistics were performed in GraphPad Prism using Tukey’s multiple comparisons test.

### 4.12. Micro-Electrode Array (MEA) recording

iPSC-MNs were plated on top of primary murine astrocytes in CytoView 48-well MEA plates (Axion Biosystems) on day 42 of differentiation. Cells were fed with BrainPhys Neuronal Medium (Stem Cell Technologies) with growth factors added. Media was changed twice a week. Spontaneous electrical field potential changes were recorded twice a week prior to media change on the Maestro MEA (Axion Biosystems). Data was recorded for 5 min, following a 1 min adjustment period. Spike (changes in electrical field potential >6 standard deviations above background), burst (minimum of 5 spikes over 1 electrode within 100 ms), and network burst (minimum of 50 spikes across at least 35% of electrodes within 100 ms) data was retrieved using the AxIS software’s Neural Metric Tool (Axion Biosystems) and averaged across 6–12 replicate wells.

### 4.13. Motor neuron survival assay

iMN cultures plated on coated 96-well plates were maintained in N3 medium with the supplements listed above, changed every other day. Hb9::RFP+ iMNs appeared between days 13–16 after retroviral transduction. RepSox, GDNF, BDNF, FGF, and CNTF were removed at day 17 and the survival assay was initiated. Longitudinal tracking was performed by imaging neuronal cultures in Molecular Devices ImageXpress once every 24 h starting at day 17. Tracking of neuronal survival was performed using AcuraStem’s proprietary image analysis pipeline that forms part of the iNeuroRx® platform. Neurons were scored as dead when their soma was no longer detectable by RFP fluorescence. All survival experiment in each was performed in two independent experiments. Equal numbers of neurons from two individual replicates from one of the trials being used for the quantification shown. All trials quantified were representative of other trials of the same experiment. Single cell longitudinal survival summary data and survival functions were then aggregated from the tracking output and graphed using GraphPad Prism. Statistical significance was evaluated by comparing test and controls by log-rank test. For each line, the survival data from 50 iMNs were selected randomly, and these data were used to generate the survival curve. Apilimod (sc-480,051) was purchased from SantaCruz Biotechnologies and dissolved in dimethyl sulfoxide (DMSO). Cells were treated in 3 μM concentrations starting day 17 (when growth factors were removed) and the treatment was replenished every 3 days with media change.

### 4.14. Drosophila genetics

All *Drosophila* stocks and crosses were kept on standard yeast/cornmeal/molasses food at 25 °C. GAL4 drivers including GMR GAL4 (eye neuroepithelium), D42 GAL4 (motor neurons), elav GAL4 (pan-neuronal) and repo GAL4 (pan-glial) were obtained from the Drosophila Bloomington Stock Center). w; Sym-UAS-ATP7 (dATP7 RNAi) and w; UAS-ATP7-GFP transgenic lines were used to knock-down and overexpress dATP7, respectively (kindly provided by Dr. Richard Burke (Hwang et al., 2014)). Human ATP7A transgenic lines were generated by injecting pBID UAS ATP7A^WT^ FLAG and pBID UAS-ATP7A^M1311V^ FLAG into w; attP40 background as described (Wang et al., 2012).

### 4.15. Larval locomotor assays

Behavioral larval assays were performed according to protocols previously described (Estes et al., 2011). In brief, wandering 3rd instar larvae were collected and allowed to acclimate to the grape juice plate for 30 sec. Afterwards, larvae were turned onto their back gently with a paintbrush, ventral side up, then the time it took for larvae to flip back (ventral side down) and make the first forward movement was recorded. A total of *N* = 33 larvae were used per genotype. Statistics were performed in GraphPad Prism 7.0 using a Kruskal-Wallis multiple comparisons test.

For drug screens, UAS ATP7A males were crossed with D42-Gal4 female virgins on fly food containing either 15 μM Apilimod or DMSO. For DMSO controls, the same volume of DMSO as the corresponding drug concentration was added (Joardar et al., 2015). Bromophenol blue was added to a final concentration of ~0.02% to ensure homogeneity. Larvae cultured on drug food or DMSO were tested for locomotor ability using larval turning assays.

## Supplementary Material

Bakkar et al, Supplemental material
