# Supplementary material for: The M1311V variant of ATP7A is associated with impaired trafficking and copper homeostasis in models of motor neuron disease
Source: Neurobiol Dis. Author manuscript; Available in PMC 2022 Nov 20. (PMC9676097; doi:10.1016/j.nbd.2020.105228)
Supplement: Bakkar et al, Supplemental material [file NIHMS1846190-supplement-Bakkar_et_al__Supplemental_material.pdf]

Supplemental Figure 1

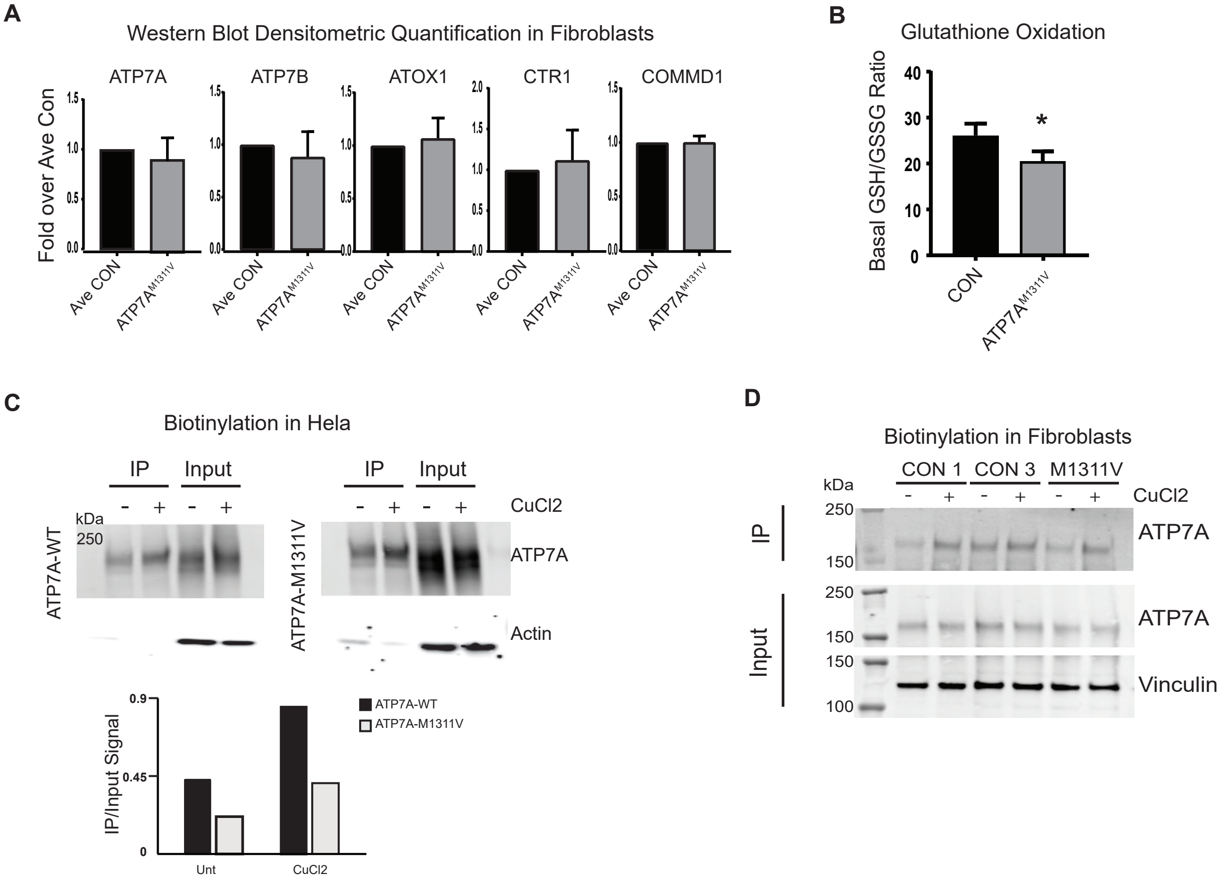

Supplemental Figure 2

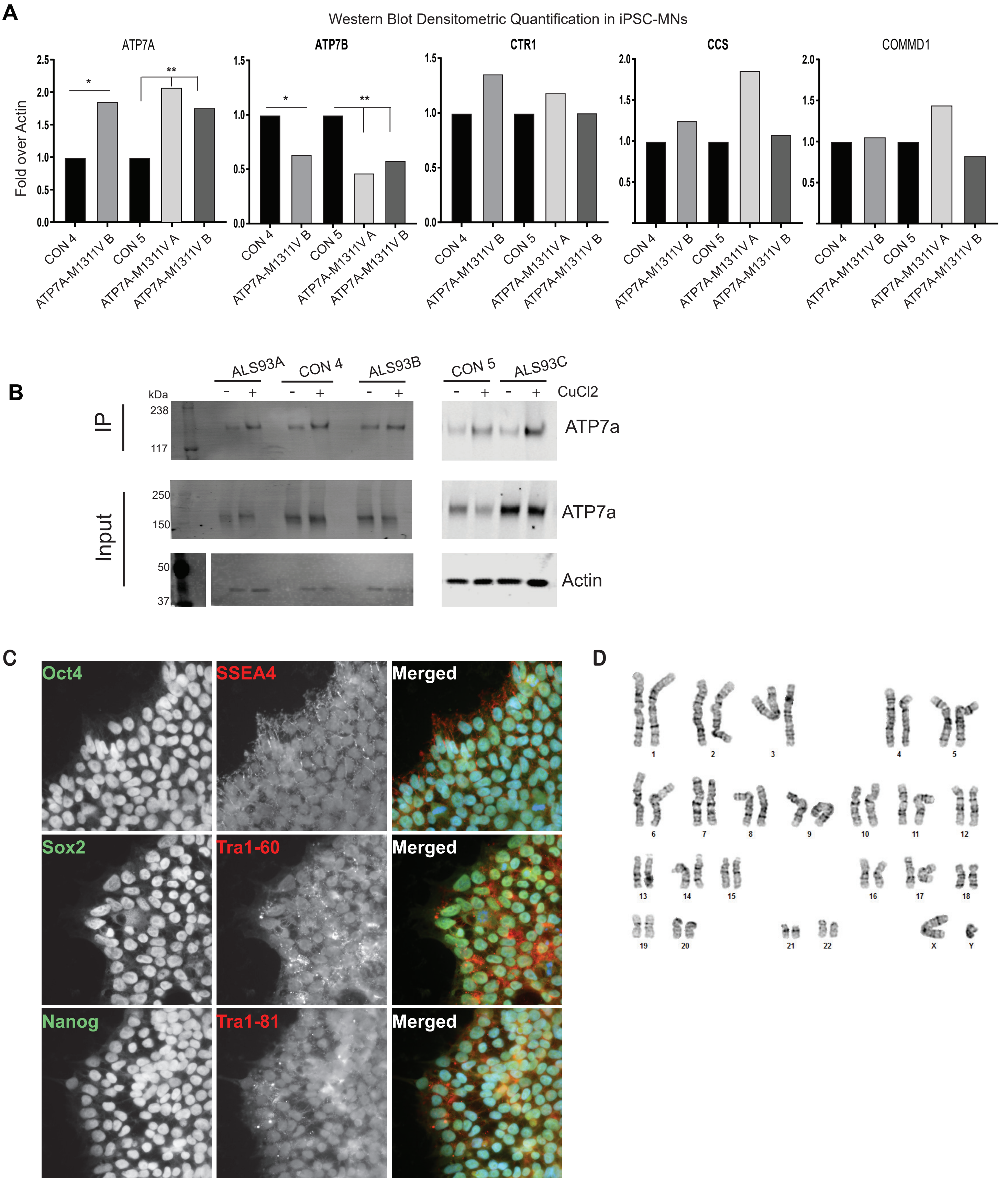

Supplemental Figure 3

A

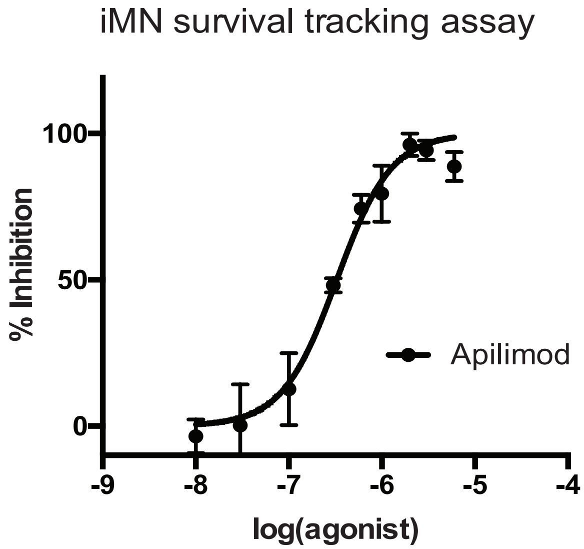

B

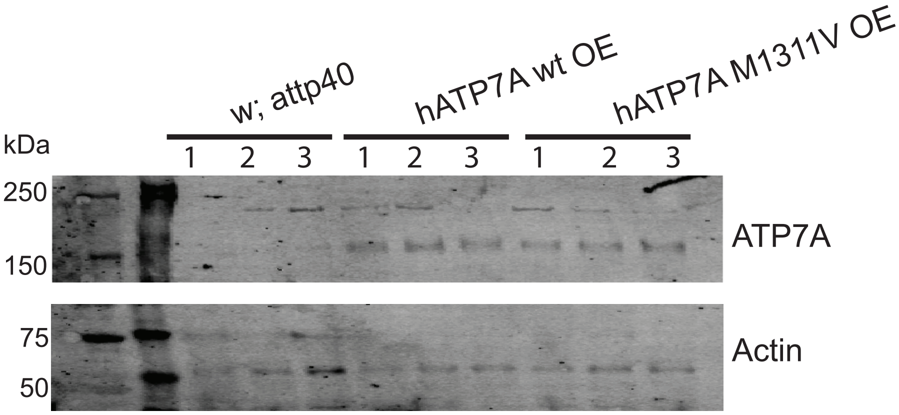

Figure Legends

Fig. S1. A) Densitometric quantification of western blot bands, normalized to vinculin. Fold changes over the average controls were calculated and averaged over three different experiments. B) Oxidized and total glutathione contents in two control fibroblast lines compared to the M1311V lines were determined. The ratio of reduced glutathione (GSH) to oxidized glutathione (GSSG) was calculated as an indicator of oxidative stress, with lower ratios indicating increased stress. C) Hela cells were transfected with pcDNA-ATP7A-WT or pcDNA-ATP7A-M1311V and 24 h later treated with Cu for 3 h. Surface biotinylation was performed and biotinylated proteins were immunoprecipitated using Avidin-conjugated beads. Western blots were then performed, probing for ATP7A in the immunoprecipitated fraction (IP), compared to the pre-IP (Input). Densitometric quantification of band intensity was performed for the IP/Input signals for each group. D) Cell surface biotinylation was performed similar to C) on two control fibroblast cell lines compared to the M1311V cells treated with 0.5 or 200 µM CuCl2 for 3 h.

Fig. S2. A) Western blots from two independent differentiations of iPSC-MNs were performed, specific protein signals were normalized to actin loading controls, and plotted as fold change over the signal from control iPSC-MNs. Asterisks denote significant differences from respective controls. B) Cell surface biotinylation was performed on two distinct differentiations of iPSC-MNs including all three $ATP7A^{M1311V}$ clones compared to two different controls. Cells were treated with 0.5 or 200 µM CuCl2 for 3 h prior to biotinylation and lysis. C) iPSCs were checked for indicated pluripotency/self-renewal markers Oct-4, Sox2, and Nanog, as well as SSEA4, Tra1-60 and Tra1-81 by immunofluorescence. D) Karyotyping was performed on the iPSCs to check for correct chromosomal numbers.

Fig. S3. A) M1311V patient-iMNs were treated with apilimod at 9 concentrations (10, 30, 100, 300, 600, 1000, 2000, 3000, 6000 nM). Cox proportional hazard model was used to generate hazard ratio from test condition vs vehicle control. Vehicle treated condition hazard ratio was defined as 0% inhibition. Hazard ratio value from the maximum response was taken as 100% inhibition. The maximum response were reached at 2–3 µM and started declining with increased concentration. Prism software was used to generate the EC50 value (log(inhibitor)vs response-variable slope (four parameters). Error bars are SEM. Data were collected from three independent experiments. B) Ventral nerve cords (VNC) were dissected from larvae expressing wild-type or mutant ATP7A and attP40 controls, lysed in RIPA and ran on SDS-PAGE to evaluate ATP7 overexpression. Genotypes are indicated. Three biological replicates were used, with each replicate/lane containing ten VNCs.
